# Designation and psychometric properties of the Short Form Postpartum Quality of Life Questionnaire (SF-PQOL): an application of multidimensional item response theory and genetic algorithm

**DOI:** 10.15171/hpp.2018.29

**Published:** 2018-07-07

**Authors:** Fariba Nikan, Mohammad Asghari Jafarabadi, Sakineh Mohammad-Alizadeh-Charandabi, Mojgan Mirghafourvand

**Affiliations:** ^1^Students Research Committee, Faculty of Nursing and Midwifery, Tabriz University of Medical Sciences, Tabriz, Iran; ^2^Road Traffc Injury Research Center, Tabriz University of Medical Sciences, Tabriz, Iran; ^3^Department of Statistics and Epidemiology, Faculty of Health, Tabriz University of Medical Sciences, Tabriz, Iran; ^4^Social determinants of health research center, Department of Midwifery, Faculty of Nursing and Midwifery, Tabriz University of Medical Sciences, Tabriz, Iran; ^5^Department of Midwifery, Faculty of Nursing and Midwifery, Tabriz University of Medical Sciences, Tabriz, Iran

**Keywords:** Quality of life, Postpartum period, Reliability, Validity, Item response theory

## Abstract

**Background: ** Utilizing multidimensional item response theory (MIRT) and genetic algorithm (GA) we aimed to design and test the psychometric properties of the short form Postpartum Quality of Life Questionnaire (PQOL).

**Methods: ** In this methodological study, 500 women aged 18 to 42 were enrolled through a multistage random sampling scheme in Tabriz, Iran. We used MIRT model and GA to identify a short form of the 40-item PQOL measure (SF-PQOL). Construct and criterion validity of the SF-PQOL was assessed by confirmatory factor analysis (CFA) and the correlation between SFPQOL scores with a 12-item short form of QOL (SF-12) and Edinburg Postnatal Depression Scale (EPDS) scores, respectively. The internal consistency, test-retest reliability and feasibility of the measure were evaluated.

**Results: ** sixteen- and 13-item SF-PQOL were identified based on MIRT and GA, respectively.The results indicate the better performance of the MIRT based 13-item SF-PQOL; Construct and criterion validity, the test-retest and internal consistency reliability, and the feasibility were confirmed in the MIRT based SF-PQOL, but not in the GA-based SF-PQOL.

**Conclusion:** The MIRT suggests a 13-item SF-PQOL with adequate content which demonstrated satisfactory validity, reliability, and feasibility. SF-PQOL could be used across the population for both research and clinical objectives.

## Introduction

The postpartum complications affect the maternal and neonatal health, profoundly. It directly influences the infant’s development,^1-3^ postpartum maternal health, including‏ Postpartum Quality of Life (PQOL). So it gained special research attention recently and could be particularly important in health promotion planning.^4^

There are few specific measures to assess PQOL in the literature, each has some limitations: Mother-Generated-Index, which is limited by its qualitative and subjective nature and the absence of cognitive skills about quality of life for women in developing countries,^4-7^ maternal PQOL, which does not address the productive health rights as well as employment status, time for rest,^8^ rural PQOL, which is limited to the only rural women’s viewpoints to assess QOL.^9^

A PQOL questionnaire was developed based on standard methods that addresses all aspects of quality of life and some aspects of reproductive health. According to the definition by the World Health Organization (WHO), this PQOL measure includes physical, psychological, and social aspects of quality of life in the postpartum period.^10^ The translation and psychometric properties of this self-administered measure were evaluated by Nikan et al in Iran.^11^ However, this measure has 40 items which is not suitable for clinical practice. In the other words, this measure should be brief enough to be more useful in both research and clinical practice. In addition, the Short Form-PQOL (SF-PQOL) should meet psychometric properties to be a reliable and valid measure, and should comprehensively measure all the content areas covered by the full PQOL. Based on an extensive search in the literature, there is no such a measure.

On the other hand, common utilization of multivariate models in the constructing health research instruments triggered an increased appeal for psychometrically inclusive and short scales. Abbreviating the scales and creating the short forms, saves the management time, while may result in a poor measure,‏ because of changing the internal structure, leading to inferior reliability, lacking discriminate between persons on the ability range, and shrinking in the test-criterion relationships.^12^ There are few approaches which have concerned the issues, we have focused on the efficiency of the genetic algorithm (GA) as a new and meta-heuristic approach and multidimensional item response theory (MIRT) item selection methodologies which have been recommended in this regard. Both of them preserve the optimal psychometric properties of the measure when constructing the short form.^12,13^ We would compare MIRT and GA to develop the short form of PQOL; The GA may assemble short versions that had the optimal properties, in comparison to the long version. MIRT can be used to appraise the psychometric properties of an existing scale, to ideally shorten the scale, and to appraise the performance of the abridged scale in the context of above-mentioned properties.^13-15^

The objectives of the study were to (1) utilize the MIRT and GA to construct the SF-PQOL, (2) to compare the MIRT and GA to the best selection between them and construct the final optimal SF-PQOL (3) to evaluate the test-retest and internal consistency reliability of the final SF-PQOL, (4) to evaluate the construct validity of the final SF-PQOL, and (5) to test the feasibility of the final SF-PQOL regarding the floor and ceiling effect. As well, we try to have the same algorithms for scoring to place the SF-PQOL scores in the same range as scored from the full PQOL item pool.

## Materials and Methods

### Study participants 

This study was methodological in nature and cross-sectional considering data collection. Information on study participants and full PQOL was published elsewhere,^11^ however, a brief description will be presented here. Participants of this study consist of 500 women aged 18 to 42 which were enrolled into the study through a multistage random sampling scheme from half of the healthcare centers of Tabriz, Iran, during November 2014 - January 2015. To conduct factor analysis for the full form with 40 items and taking into account at least 5 subjects per item,^16^ a sample size of 200 was required. However, multiplying by 2, to account for the design effect (=2) in the multistage sampling design of the study,^17^ and to conduct required analyses on separate sub-samples (calibration and validation sub-samples), the sample size increased to 500. To address the item response theory analysis, Tsutakawa and Johnson recommend a sample size of about 500 for truthful parameter estimates.^18^ However, other studies showed that 200 or fewer observations can be adequate.^19,20^ Also, this sample size was sufficient for using GA in our data.^21^

The inclusion criteria were: having a singleton, healthy, and term newborn weight over 2500 g, guidance school or higher education, being Iranian and having access to the phone or mobile. In Iran, the second month vaccination of all children is conducted in public health centers/posts, and a list of individuals referred for vaccination and also their phone number is recorded in a specified notebook. We used this information to achieve eligible participants. Among the 42 health centers and 33 health posts in Tabriz, 22 health centers and 15 health posts were randomly selected, respectively. The samples were proportionately selected based on the code of referring to the selected centers/posts for giving the second-month vaccination. In each health center/post, the registered mothers, who were in the 60-67 postpartum days, were selected randomly. The potentially eligible mothers were invited to refer the health centers/posts to participate into the study. After a brief explanation of the objectives and procedure of the study, eligible subjects were asked to complete a set of paper-based questionnaires.

### Measures 

Concerning optimal properties (to be a useful measure for both research and clinical setting to screen quality of life problems, and to be cost-effective and covering all aspects of quality of life), we choose the full PQOL after permission from the developers.^10^ The full PQOL is a 40 item self-administered measure that comprises of 4 dimensions; physical functioning, child care, psychological functioning and social support with 8, 12, 8 and 12 number of items, respectively. Each item is answered with a 5-point Likert scale, to assess the intensity such as: ‘‘(1) Not at all, (2) Slightly, (3) Moderately, (4) Very, (5) Extremely’’, the frequency, such as: ‘‘(1) Never, (2) Rarely, (3) Sometimes, (4) Often, (5) Always’’ and evaluation such as: ‘‘(1) Very dissatisfied, (2) Dissatisfied, (3) Neither satisfied nor dissatisfied, (4) Satisfied, (5) Very satisfied’’. The normalized scores were computed which ranged over a 0-100 interval with 0 and 100 indicating the poorest and the best PQOL score respectively. Along with full PQOL, demographic questionnaire, Edinburgh Postnatal Depression Scale (EPDS)^22^ and Short Form Health Survey (SF-12)^23^ were given to the mothers. The psychometric properties of the PQOL were evaluated by Nikan et al. However, briefly, the PQOL indicate a good internal consistency (Cronbach’s alpha ranged over 0.70-0.88), and a good test-retest reliability (intraclass correlation coefficients [ICCs] ranged over 0.87-0.92). The construct validity as assessed by exploratory factor analysis (EFA) and confirmatory factor analysis (CFA), indicate an acceptable fit for a 4-factor solution model. The strong correlation between the PQOL and SF-12 supported the criterion related validity of the measure. Finally, the discrimination ability of the PQOL was confirmed, differentiating the EPDS-based depressed and un-depressed women.^11^ Both EPDS and SF-12 measures can discriminate well between known-groups (supporting their discriminant validity) and showed good internal consistency reliability (Cronbach’s alpha >0.70).

### Statistical analyses

The data were expressed by mean (SD) and by frequency (%) for the numeric and categorical variables, respectively. The normality assumption of the numeric variables was assessed by skewness and kurtosis indices; the values greater than 3 and 10 in skewness and kurtosis, suggest a serious problem in normality, respectively. Above mentioned analyses were performed on entire samples, however, for succeeding analyses, data were randomly divided into the test (n = 250) and the validation (n = 250) samples and will be mentioned where it would be used.

### Genetic algorithm

GA, introduced by Holland, relies on the fundamental Darwinian evolution principles of selection, crossover, and mutation.^12^ GA was utilized to abbreviate the PQOL measure. We applied the default optimization offered in the R package *GAabbreviate.*^24^ The cost reduction function was:

*Cost = Ik* + 1 – *R*^2^

where *I* and *k* denote a fixed item cost and the number of items reserved by the GA in each iteration, respectively. *R*^2^ is the amount of variance explained for by a linear combination of individual item scores for all subscales. By shifting the values of *I*, we can place higher or less stress on the shortness of the measure compared to its inclusiveness.^25^ High and low values of *I* would lead to a relatively short and a comparatively longer measure, respectively. GA aims to reduce the redundancy within a scale, and so to abbreviate the items that the best capture the scale of interest.^12,26^ In the present study, we change *I* to reach the maximum amount of the R^2^ and hence the minimum amount of the cost, which leads to the SF-PQOL with 16 items. We set the GA in binary type, a population size of 100, number of generations of 1000, elitism of 5, crossover probability of 0.8 and mutation probability of 0.1.

### Multidimensional item response theory 

IRT is a set of latent variable techniques specifically designed to model the interaction between a participant’s ability/latent trait, with item level stimuli (difficulty, discrimination, guessing, etc). The Unidimensional IRT, model each item with only one latent trait. While the MIRT, model each item with more than one latent trait, due to its added flexibility. In this paper, considering the 4 latent traits in the questionnaire,^11^ the MIRT with graded response models (GRMs) was utilized to model ordinal items.^13,27,28^ R package “mirt” was used to fit‏ GRMs. “mirt” is an open-source software, useful for real data analysis and research. “mirt” provides multidimensional estimation techniques‏. We perform the exploratory MIRT models with Metropolis-Hastings-Robbins-Monro (MHRM), quasi-Monte Carlo EM (QMCEM) estimation method^13^ in the test sample. To reach the best number of the items, we used Akaike information criteria (AIC), Bayesian information criteria (BIC), corrected Akaike information criteria (AICc), sample size adjusted Bayesian information criteria (SABIC), which are based on the -2log Likelihood index. Smaller values of the criteria indicate a better fit of model to the data. Additionally, the fit of the model with the best number of items has also been examined and confirmed in the validation sample. The adequacy of the model was assessed by the goodness of fit indices. Reasonable values are: chi-square/df <2, root mean square error of approximation (RMSEA) <0.05, standardized root mean square residual (SRMR) <0.05 and also, comparative fit index (CFI) > 0.95, Tucker-Lewis index (TLI) >0.95.^16,29^

### Procedure to conduct the MIRT in constructing the short form

We used the item fit analyses with the S-χ^2^ statistic to identify the SF-PQOL items. This statistic compares observed and expected response frequencies under the used MIRT model, and measures the differences between these frequencies. The significant S-χ^2^ statistic shows an item with model deviation so that removing these items, would lead to a model with better fit.^15,30^ We consecutively removed the items with the least significance Hochberg adjusted probability in each step. Finally, we reached the model with 13 items. After which there was no significant S-χ^2^ statistics.

### Preliminary validation of the short form 

The validation of the full PQOL in all types including scale translation validity, linguistic edit, content validity, face validity, construct validity, discriminant and criterion validity have comprehensively been assessed and the scale properly been modified.^11^ However, to assess the psychometric properties of the SF-PQOL, some procedures are detailed.

### Construct validity 

To assess the construct validity and to compare the results between full measure and SF-PQOL, we conducted CFA for both measures in the validation sample. The CFA was conducted by weighted least squares estimation method. The covariance matrix and asymptomatic covariance matrix were considered as the input and weight matrix, respectively. The adequacy of the model was assessed by the goodness of fit indices. Reasonable values are: chi-square/df <2, root mean square error of approximation (RMSEA) <0.05, standardized root mean square residual (SRMR) <0.05 and also, comparative fit index (CFI) >0.95, Tucker-Lewis index (TLI) >0.95.^16,29^

### Criterion validity

The SF-12 has been established as a standard tool to assess the quality of life in the Iranian population.^23,31^ Additionally, studies have shown a reverse correlation between quality of life and depression in postpartum women^32^ as measured by EPDS. In this study, these measures were used to evaluate the criterion validity of both full and SF-PQOL measures. The Pearson’s correlations between the SF-12 total and domains’ scores with full and SF-PQOL were tested. Values less than 0.1, between 0.1 and 0.3, between 0.3 and 0.5, and greater than 0.5 indicated non-significance, poor, medium and strong correlations, respectively.^33^

### Reliability

Internal consistency was assessed by Cronbach’s alpha coefficient.^34^ Alpha coefficients higher than or equal to 0.60 were considered acceptable. Test-retest reliability was assessed by completing the questionnaire two times within 2 weeks by the same 30 randomly selected women. ICC was computed to test the stability over time. ICCs ≤ 0.4 were considered poor to fair, ICCs: 0.41-0.60 moderate, ICCs: 0.61-0.80 good and ICCs >0.80 excellent.^35^

### Feasibility

To assess the feasibility of the measures, the percentages of possible minimum and maximum scores were computed as floor and ceiling effects respectively.

All statistical analyses for above mentioned properties were performed using STATA 14. *P* values less than 0.05 were considered as significant.

### Scoring system of SF-PQOL

We tried to have the same algorithms for scoring to place the SF-PQOL scores in the same range as scored in the full PQOL. Therefore we utilized the normalized score based on the following formulae:

Normalized score = (raw score – minimum) ÷ (possible range) × 100

Which project the item responses in the range of 0-100. The scores of the total and subscales in the full and SF-PQOL were created based on the average over the related items. The scores obtained by this formula are compatible with the short and full forms.

## Results

All data were collected during a 2-month period in 2014-2015 comprising 500 pregnant women. The missing data were imputed using the multiple imputation method. For imputation, the PQOL items were used both as predictor and imputation in an expectation-maximization (EM) algorithm. The missing data comprised less than 5% of all the items.

The mean age of the participants was 28 (SD 5) years, half of them had secondary education. About half (53%) of the women were primiparous, and 372 of them (74%) had a cesarean section (Table 1).

The item content and percentages of responses for full PQOL is presented in Table 2.

### Splitting data to test and validation samples

The descriptive results in Tables 1 and 2 were produced in the whole sample. However, for the succeeding analyses, the data were randomly divided into a test (n = 250) and a validation (n = 250) sample which would be indicated where it would be used.

### Results of genetic algorithm

**Figure 1:**
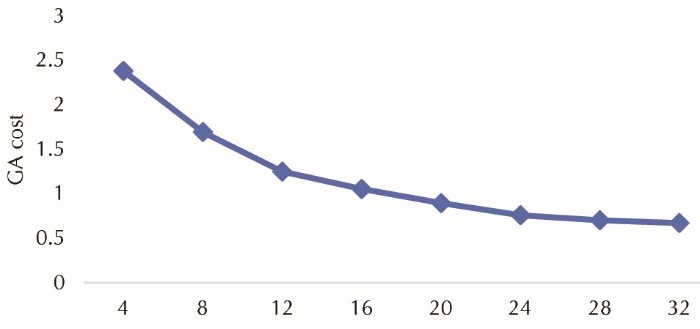
Genetic algorithm (GA) cost versus item number.

We first fit the models with 4, 8, 12…and 32 items (considering the 4 subscales in the PQOL, we choose 4 and multiplies of 4). A graph of cost versus the number of items, led to a set of 16 items (Figure 1). In this point, the cost reached the minimum value (cost=1.054) compared to the models with 12, 8 and 4 items. The mean convergent correlation in training and validation models were 0.87 and 0.88, respectively.

### GA-selected items

We choose a 16-item solution: a 4-dimension solution each contains 4 items. The subscales and their remained items were:

Child care: PQOL1, PQOL5, PQOL6, and PQOL8

Physical functioning: PQOL9, PQOL16, PQOL18, and PQOL20

Psychological functioning: PQOL21, PQOL25, PQOL26, and PQOL28

Social support: PQOL29, PQOL32, PQOL33, and PQOL34

### Results of MIRT 

**Figure 2:**
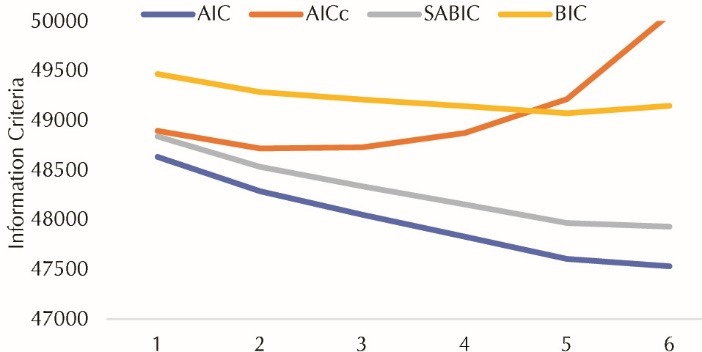
Information criteria versus dimension number.
AIC: Akaike information criteria, BIC: Bayesian information criteria, AICc: corrected Akaike information criteria, SABIC: sample size adjusted Bayesian information criteria.

To determine the optimal number of factors, we fit the models with 1, 2 …and 6 factors. A graph of Information criteria (Figure 2) versus the number of items, led to a model with 4 factors. In this point, the information criteria reached the minimum value approximately, compared to the models with 5 and 6 factors. In addition, the theoretical support of 4-factor solution, led us to choose the model with 4 factors.

### Initial MIRT model with 4 factor in the full PQOL

In general, the 4-factor solution MIRT model for the full PQOL had not a good model fit (χ^2^_(508)_ = 1282.4, *P* < 0.001, χ^2^ /df = 2.5 > 2, RMSEA = 0.055 >0.05 and 90% CI = (0.052-0.059), CFI =0.899 < 0.95, TLI = 0.869 < 0.95, SRMR =‏ 0.056 > 0.05). Additionally, there were many items with the commonalities < 0.2. Also, there were many items with large S-χ^2^ values and hence Hochberg adjusted *P* value of S-χ^2^ <‏ 0.05, indicating the items which were candidates to be deleted from the subscales. In this model, the estimates of discriminating parameter ranged over <0.01–1.96. The difficulty parameters for the 40 items reflect a sizable range of underlying PQOL (-6.9 to 10.7).

### MIRT-selected items

The item fit analyses were used along with the S- χ^2^ statistic to identify a shortened instrument. The significant S- χ^2^ statistic shows an item which produces a model deviation according to the Hochberg’s adjusted *P* value. The items with the least adjusted significance probability in each step were consecutively removed. Finally, the model with 13 items was achieved, after which there was no significant S- χ^2^ statistic (Table 3).

The remained items to construct SF-PQOL sub-scales were:

- Physical functioning: PQOL9, PQOL17, PQOL18, and PQOL20

- Child care: PQOL2, PQOL7, PQOL14, and PQOL22

- Psychological functioning: PQOL27, PQOL28, and PQOL31

- Social support: PQOL29 and PQOL30

The results of MIRT factor analysis indicate correlated dimensions (correlation coefficients ranged over 0.367 - 0.494). The parameter estimates from the fully specified MIRT model showed the discriminating parameter estimates ranged over <0.01–2.57. The difficulty parameters for the 13 items reflect a sizable range of underlying PQOL (-6.17 to 7.54) (Table 3).

### Comparing the model fit based on GA-selected and MIRT-selected items in validation data set

The CFA based 4-factor solution on GA-selected items did not fit well on the validation data set (χ^2^_(656)_ = 8392.4, χ^2^/df = 12.8, RMSEA = 0.154 > 0.05 and 90% CI = (0.151-0.157), CFI < 0.1, TLI < 0.0, SRMR + 0.193 > 0.05). Also, the results showed no satisfactory internal consistency for subscales (α equals to 0.15, 0.53, 0.55 and 0.64) and the total score (α equals to 0.48), however, a good level of stability reliability was observed for subscales (ICC equals to 0.85, 0.89, 0.78 and 0.89) and the total score (ICC 0.93).

In the other hand, in the final step, MIRT had a good model fit on the validation sample data, though the SRMR was so close to the nominal value (χ^2^_(44)_ = 53.2, P=0.162, χ^2^ /df = 1.2 < 2, RMSEA = 0.029 < 0.05 and 90% CI = (0.052-0.059), CFI =0.991 > 0.95, TLI = 0.977 > 0.95, SRMR =‏ 0.053 > 0.05).

Since the results showed a better model fit for MIRT compared to the GA, therefore, we choose the MIRT-based SF-PQOL as our optimal measure and subsequent analyses have been just conducted on this measure.

### Preliminary validity, reliability and feasibility of the MIRT based SF-PQOL

The results of the validity and reliability presented in the following sections are obtained in the validation data set and a data set consisting of 30 randomly selected women was used for test-retest reliability.

The CFA results for the SF-PQOL in the validation data set showed an acceptable fit, but the results were suboptimal in the full PQOL (Table 4).

The results showed a satisfactory internal consistency for subscales (α ranged over 0.68-0.85) and the total score (α > 0.7), and a good stability reliability for subscales (ICC ranged over 0.86-0.88) and the total score (ICC > 0.7).

The percentage of the ceiling and floor scores were (0% and 2.0%), (0% and 0.8%), (0% and 0.4%), (1.6% and 4.4%), and (0% and 0%) respectively, for subscales of physical functioning, childcare, psychological functioning and social support and PQOL total score (all less than 15%), indicating excellent level of feasibility of the SF-PQOL. Values of skewness (<3) and kurtosis (<10) measures in total and sub-scales’ scores indicated the normality assumption of the scores (Table 5).

Negative and significant correlations were observed between the sub-scales’ and total scores of PQOL, with the EPDS score in both full and SF-PQOL. Alternatively, positive and significant correlations were observed between the sub-scales’ and total scores of PQOL, with the scores of mental and physical subscale and the total score of the SF-12. Additionally, a positive and strong correlation (r = 0.75) was observed between the full and SF-PQOL total scores (Table 6).

## Discussion

The results of the study supported the calibration of final version of the SF-PQOL abbreviated from the full PQOL item pool utilizing the MIRT and the GA. Construct and criterion validity, test-retest and internal consistency reliability and the feasibility of the SF-PQOL were confirmed by a validation data set. SF-PQOL demonstrated satisfactory validity, reliability, and feasibility. Thus SF-PQOL may be used across the population for both research and clinical objectives.

### Rationale behind utilizing the MIRT and the GA

To the best of our knowledge, this is the first study to apply the MIRT and the GA to the PQOL instruments in Iran, evaluating the ability of the questionnaires’ items to identify individual latent traits. Globally, this is the first study utilized the MIRT and the GA in constructing a short form of the PQOL. The IRT especially the MIRT as well as the GA were used by many studies to construct the short form of health outcomes.^12,14,36-39^ Additionally IRT has been recommended for selecting the items that are most informative to develop tailored instruments.^40^ The MIRT was very useful and informative in constructing a well-organized short form instrument to be working in both clinical practice and research; wide enough to be research based and brief enough to be practical. Although the SF-PQOL is shorter than most commonly utilized PQOL measures, it has maintained adequate content. The satisfactory results of IRT were reported in other studies.^14,41^ Graded response modeling of the IRT was used considering the ordinal nature of the responses which is a recommended modeling.^14,28^ Although it is suggested that this methodology may not be tailored for all measures and should be used along with other traditional psychometric methods.^42^ Especially there are some methodological issues in applying the IRT methodology such as the underlying assumptions in unidimensional IRT which should be fulfilled to have the valid results. Although many of the issues have been solved in MIRT.^13^

### Motivation to utilize the SF-PQOL

As the advantage of the SF-PQOL is its importance to evaluate the quality of life in postpartum women. The SF-PQOL saves time and costs in this evaluation, because of its short form. One of the important concern about consultation on postpartum and postnatal care is the length of time that patients have to consult with their caregiver.^43^ The length of time for consultation may be insufficient for patients to adequately describe their health status. Therefore, one of the potential uses of SF-PQOL in clinical practice may be its ability to evaluate the PQOL in a short time period (3-4 minutes). This is well within the typical range of consultation time in general practice. Additionally, due to its self-administered nature, saving time and cost, it can be utilized in clinical settings by midwives, doctors, and nurses who are involved in postpartum care. It can also be properly used in research settings because of its item content and information coverage.

### Constructing SF-PQOL utilizing MIRT

The results of this study were in the line of the previous study: at first, we evaluated the psychometric properties of the full PQOL.^10,11^ Then, to identify a short form, the GA and item fit statistics from the MIRT were used,^12,13^ the results indicate the optimality of the MIRT. Hence based on the MIRT, the SF-PQOL was constructed containing the 30% of the entire PQOL item pool. The SF-PQOL domains cover the content areas found in the widely used measures of quality of life according to the WHO’s definition; including child care, physical, psychological, and social aspects, which have to be addressed.^44^

### Construct validity of the SF-PQOL

The CFA of SF-PQOL was confirmed based on the goodness of fit indices; this is consistent with related studies.^10,11^ Additionally, the CFA was assessed for full PQOL in the validation sample; the results showed superior supporting of CFA in the SF-PQOL as compared to the full PQOL. The construct validity of the SF-PQOL was confirmed in the validation data.

### Reliability of the SF-PQOL

The SF-PQOL slightly outperformed the full PQOL in the terms of internal consistency reliability assessed by alpha, however, the ICC values showed a good stability reliability of the SF-PQOL total score and subscales. The results are consistent with other studies in full PQOL.^10,11^

### Criterion-related validity of the SF-PQOL

Negative and significant correlations were observed between the sub-scales’ and total scores of the PQOL, with the EPDS score in both SF-PQOL and full PQOL (however the correlation between social support and EPDS in original form was weak). This is in the line with many studies which showed that increased postpartum QOL is associated with a lower EPDS score.^45-47^ On the other hand, positive and significant correlations were observed between the sub-scales’ and total scores of the PQOL with the scores of mental and physical subscales and the total score of the SF-12, (except for the social support domain in original form PQOL). This is consistent with other studies which showed that improved postpartum QOL is correlated to higher SF-12.^11,48^ Additionally, a positive and strong correlation (r = 0.75) was observed between the full and the SF-PQOL. The similar amounts of correlation were observed between short and original forms of health outcomes in other studies.^14,41^ As expected, high scores on the SF-PQOL along with the SF-12 and its reverse relation with EPDS, was an indication of the satisfactory quality of life,^45-47^ which finally confirmed the criterion validity of the SF-PQOL. Additionally, relatively the similar amounts of correlations were observed among the scores in the full and the SF-PQOL with other scales.^11^

### Feasibility of the SF-PQOL

The percentage of floor and ceiling effect were all less than 15% in the PQOL total score, physical functioning, child care, psychological functioning and social support subscales in the SF-PQOL, indicating the feasibility of the SF-PQOL. This is consistent with the previous study on this measure.^11^

### Strengths and limitations

This is the first study which introduces the short form of a specific measure of QOL in postpartum women utilizing the MIRT and the GA. Our study has several limitations; First, differences in the values and cultural systems among rural and urban areas may limit the generalizability of the results to the country’s rural areas. Second, all subjects were Tabriz residents, the fifth largest city in Iran with another language (Azeri), therefore, studies on the reliability and validity of the SF-PQOL in other parts of Iran and in other sub-groups of women (with different languages ​​and cultures) are recommended.

## Conclusion

The evidence presented from this study suggested that we successfully achieved our goals of developing a brief measure of the quality of life; a 13-item short form and a relatively precise measure of the PQOL with good content coverage. Also, the study confirmed the psychometric properties of the SF-PQOL in the Iranian women. Utilizing this measure can solve the obstacles in evaluating the postpartum women’s quality of life in both clinical and research settings. In addition, it is recommended to be used by those involved in postpartum care, such as midwives in health centers. It appears that the SF-PQOL can facilitate the postpartum care and help evaluate the women’s quality of life and identify potential problems in this important period.

## Ethical approval

First, the protocol of the study was approved by the institutional review board of Tabriz University of Medical Sciences (ethical approval code: IR.TBZMED.REC.1395.290; date: 12, June 2016). In addition, participants were informed of the research procedure, comprehensive information on the objectives; they also were informed about confidentiality, privacy, the right to end their participation and benefits. A signed informed consent form was obtained from all participants before data collection. All questionnaires were anonymous, and files that included participants’ contact were shredded after all data were collected. Only the research-related personnel could access and use the data. The study was conducted according to the world medical association Declaration of Helsinki.

## Competing interests

The authors have no conflicts of interest to disclose.

## Authors’ contributions

All authors read and approved the final manuscript. FN, MAJ and SMACH conceived of the study and participated in the design and data collection. MAJ, SMACH and MMV participated in the data analyses and MS preparation.

## Acknowledgments

Hereby, we greatly appreciate the cooperation provided by research vice chancellor and health deputies of the Tabriz University of medical sciences, all personnel of health centers in Tabriz and all participants in this research. This project was funded by research chancellor, Tabriz University of Medical Sciences, Tabriz, Iran (Grant No:1395.290).

**Table 1:** Socio-demographic profile of the participants (n = 500)

**Characteristics**	**No. (%)**
Age (y)	
˃20	27 (5.4)
20-34	423 (84.8)
≥35	50 (9.8)
Education	
Secondary (6-8 y)	84 (16.8)
Senior high school (9-12 y)	250 (50.0)
College (+13 y)	166 (33.2)
Occupation	
Student	18 (3.6)
Housewife	425 (58.0)
Employed	57 (11.4)
Parity	
First	267 (53.4)
Second	210 (42.0)
Third and over	23 (4.6)
Type of recent delivery	
Vaginal	128 (25.6)
Caesarean section	372 (74.4)
EPDS	
Depressed (Mean score ≥14)	118 (23.6)
Non-depressed (Mean score ≤13)	382 (76.4)

Abbreviation: EPDS, Edinburgh Postnatal Depression Scale.

**Table 2:** Item content and percentages of responses for 40-item PQOL in total sample (n = 500)

**Items**	**Item content**	**1**	**2**	**3**	**4**	**5**
PQOL1	Do you worry that your‏ child will fall sick?	18.6	19.4	39.6	10.0	12.4
PQOL2	How satisfied are you with your child’s health?	2.8	2.4	10.6	47.6	36.6
PQOL3	Do you worry that your child will have an accident?	13.0	19.0	38.2	13.0	16.8
PQOL4	How much do you take pains to prevent an accident to your child?	39.0	36.4	15.2	5.6	3.8
PQOL5	Do you worry about the nutrition of your child?	45.4	17.6	16.6	7.8	12.6
PQOL6	Do you worry that your child is not smart?	10.6	14.4	19.8	17.4	37.8
PQOL7	Do you think that your breast milk is enough for your child?	7.4	10.2	25.2	37.6	19.6
PQOL8	How satisfied are you with current feeding?	1.2	6.0	14.6	50.6	27.6
PQOL9	Do you worry about unexpected pregnancy?	41.2	14.0	18.6	10.0	16.2
PQOL10	How much are you bothered by contraception?	10.6	12.2	29.2	15.8	32.2
PQOL11	How satisfied are you with your sleep?	9.6	22.8	30.8	30.6	6.2
PQOL12	Do you have enough time to rest?	7.0	19.4	37.0	27.6	9.0
PQOL13	How easily do you get tired?	9.4	23.0	47.0	18.2	2.4
PQOL14	How satisfied are you with the energy that you have?	5.6	17.0	32.0	39.8	5.6
PQOL15	Does physical pain influence your daily life?	5.2	20.4	36.8	21.8	15.8
PQOL16	How much do you think that your physical health has been affected by childbirth?	4.6	22.2	36.2	26.2	10.8
PQOL17	How much conflict do you feel between child care and work?	8.8	18.6	38.6	25.6	8.4
PQOL18	Has your child caused you to be distracted and worried at work?	8.4	25.0	25.4	28.2	13.0
PQOL19	How satisfied are you with the way your body looks?	13.8	22.6	25.0	32.8	5.8
PQOL20	Do you feel blue by your looks?	12.6	12.2	26.8	17.4	31.0
PQOL21	How much confidence do you have in caring for your baby well?	0.8	3.0	26.0	45.8	24.4
PQOL22	How much child care skill do you think you have?	0.8	2.4	39.6	41.4	15.8
PQOL23	Are you interested in your child?	0.0	0.0	0.2	4.8	95.0
PQOL24	Are you willing to look after your child?	1.8	2.2	6.0	9.0	81.0
PQOL25	Do you regret having had this child?	0.6	2.8	8.2	8.2	80.2
PQOL26	Is caring a baby hard for you?	1.4	9.0	32.0	17.4	40.2
PQOL27	Are you happy being a mother?	88.4	6.8	3.8	0.4	0.6
PQOL28	How much fun is your life after having this child?	1.4	4.4	17.8	40.4	36.0
PQOL29	Do you have enough contact with the outside world?	5.8	13.2	51.6	23.0	6.4
PQOL30	Do you see enough of your neighbors?	2.2	4.8	33.4	30.2	29.4
PQOL31	What do you think your husband’s attitude is towards you?	1.0	1.2	19.2	41.6	37.0
PQOL32	How close is the relationship between you and your husband?	1.6	3.6	22.2	35.4	37.2
PQOL33	How much help do you get caring for your child?	5.2	15.8	43.2	27.6	8.2
PQOL34	How much help do you get doing household chores?	2.8	9.6	31.6	24.8	31.2
PQOL35	How clean is your house?	9.4	37.4	46.8	5.4	1.0
PQOL36	How satisfied are you with your housing situation	3.2	5.0	29.0	47.2	15.6
PQOL37	Is the money that yourself can decide how to spend enough?	4.2	29.6	34.0	13.8	18.4
PQOL38	Do you worry about your finances?	25.2	22.4	31.4	11.6	9.4
PQOL39	How satisfied are you with your living environment, including pollution, noise, climate, and location?	2.4	7.0	29.2	48.8	12.6
PQOL40	How satisfied are you with the transportation available to you?	21.0	45.2	20.4	7.0	6.4

Abbreviation: PQOL: Postpartum Quality of Life For items 1, 3, 4, 5, 6, 9, 12, 15, 23, 24, 25, 26, 27, 38, the responses were; 1: Never, 2: Rarely, 3: Sometimes, 4: Often and 5: Always. For items 2, 8, 11, 14, 19, 36, 39, 40, the responses were; 1: Very Unsatisfied, 2: Unsatisfied, 3: Neither satisfied nor unsatisfied, 4: Satisfied, 5: Very satisfied. For items 7, 37, the responses were; 1: Is not enough at all, 2: Is not enough, 3: Sometimes, 4: Is enough, 5: Is always enough. For items 10, 13, 16, 17, 18, 20, 21, 22, 28, 29, 30, 32, 33, 34, 35, the responses were; 1: Not at all, 2: Little, 3: Moderately, 4: Very, 5: Too much. And for item, 31 the responses were; 1: Very bad, 2: Bad, 3: Neither bad nor good, 4: Good, 5: Very good.

**Table 3:** MIRT based SF-PQOL scales, items and model results

**PQOL subscales**	**PQOL items**	**MIRT factor loading**	**Item fit** ***P*** ** value**	**Discriminating parameter**				**Difficulty parameters**			
				**a1**	**a2**	**a3**	**a4**	**d1**	**d2**	**d3**	**d4**
Physical functioning	PQOL2	-0.716	0.824	1.20	0.08	1.21	0.29	4.76	4.04	2.48	-0.82
	PQOL7	-0.395	0.943	0.52	0.03	0.52	0.05	2.75	1.72	0.34	-1.56
	PQOL14	-0.392	0.208	1.43	0.03	0.55	0.27	3.68	1.73	-0.23	-3.80
	PQOL22	-0.430	0.391	0.84	0.05	0.55	0.04	5.26	3.82	0.36	-1.99
Child care	PQOL9	-0.378	0.827	0.43	0.08	0.08	0.47	1.78	1.13	0.24	-0.38
	PQOL17	-0.766	0.290	1.24	0.19	0.09	1.34	3.52	1.04	-1.49	-3.40
	PQOL18	-0.651	0.184	1.34	0.01	0.05	1.02	2.73	0.51	-1.04	-3.33
	PQOL20	-0.409	0.695	0.98	0.02	0.11	0.48	0.99	0.09	-1.35	-2.31
Psychological functioning	PQOL27	0.637	0.879	1.27	0.05	0.69	0.3	-2.71	-3.86	-5.62	-6.17
	PQOL28	-0.859	0.505	2.57	0.21	0.84	0.92	7.54	5.34	2.36	-1.24
	PQOL31	-0.396	0.992	1.54	<0.01	<0.01	<0.01	5.67	4.78	1.79	-0.76
Social support	PQOL29	0.766	0.441	1.57	1.73	0.18	<0.01	4.68	2.58	-1.56	-4.43
	PQOL30	0.767	0.482	1.02	1.53	<0.01	<0.01	5.29	3.72	0.59	-1.34

Abbreviation: MIRT, Multidimensional Item Response Theory; PQOL, Postpartum Quality of Life; SF, Short Form.

**Table 4:** Results of CFA fit indices of the full and SF-PQOL in the validation sample (n = 250)

**Measure**	**χ** ^ 2 ^	***df***	**χ** ^ 2 ^ **/** ***df***	**RMSEA (90% CI)**	**SRMR**	**CFI**	**TLI**
Full PQOL	1897.14	740	2.56	0.043 (0.037; 0.048)	0.067	0.870	0.860
SF-PQOL	90.05	71	1.27	0.033 (0.001; 0.052)	0.042	0.971	0.963

Abbreviation: CFA, confirmatory factor analysis; df, the degrees of freedom; χ^2^/*df*, normed chi-square; RMSEA, root mean square error of approximation, SRMR; root mean square residual, CFI; comparative fit index, TLI; Tucker-Lewis Index; PQOL, postpartum quality of life. All item scale relationships were statistically significant *P <* 0.001

**Table 5:** Summary statistics, Cronbach’s a and ICC for the SF-PQOL in validation sample (n = 250)

**PQOL Subscales and total**	**Mean**	**SD**	**Skewness**	**Kurtosis**	**Cronbach’s α**	**ICC (95% CI)**	**Floor %**	**Ceiling %**
Physical Functioning	65.9	15.3	-0.12	2.71	0.85	0.88 (0.73-0.94)	0.0	2.0
Child care	50.2	21.1	-0.16	2.55	0.71	0.86 (0.71-0.94)	1.2	0.8
Psychological Functioning	52.8	12.2	-0.29	3.86	0.87	0.82 (0.72-0.94)	0.0	0.4
Social support	61.2	20.6	-0.36	3.25	0.68	0.86 (0.71-0.94)	1.6	4.4
Total QOL	57.4	9.8	-0.02	2.86	0.73	0.82 (0.62-0.92)	0.0	0.0

Abbreviation: ICC, intraclass correlation coefficient; SD, standard deviation; PQOL, postpartum quality of life. The normalized score ranged over 0–100 total and all subscales, with lower values indicating lower quality of life.

**Table 6:** Correlation among PQOL subscales with total PQOL score, EPDS, and SF12 subscale and total score in the SF-PQOL and full PQOL in validation sample (n = 250)

**SF-PQOL subscales and total**	**Full PQOL**	**EPDS**	**Mental QOL**	**Physical QOL**	**SF12**
SF-Child care	0.59	-0.47 (-0.34)	0.37 (0.28)	0.34 (0.29)	0.42 (0.33)
SF-Physical Function	0.64	-0.42 (-0.58)	0.35 (0.52)	0.32 (0.55)	0.39 (0.62)
SF-Psychological Function	0.66	-0.43 (-0.29)	0.26 (0.16)	0.28 (0.17)	0.31 (0.19)
SF-Social support	0.44	-0.31 (-0.56)	0.31 (0.37)	0.32 (0.41)	0.36 (0.44)
SF-PQOL	0.75 ^a^	-0.54 (-0.63)	0.40 (0.49)	0.36 (0.52)	0.44 (0.58)

Abbreviation: EPDS, Edinburgh Postnatal Depression Scale; PQOL, postpartum quality of life; SF: Short Form; SF12: Short Form12 Quality of Life. Correlation values in the full PQOL are presented inside of the parenthesis and the correlation values in the SF-PQOL are presented outside of the parenthesis. All correlations were significant (All *P* < 0.05). ^a^ Significant correlation was observed between SF-PQOL full form (r = 0.75, *P* < 0.001).
